# Chronic multiscale imaging of neuronal activity in the awake common marmoset

**DOI:** 10.1038/srep35722

**Published:** 2016-10-27

**Authors:** Yoshiyuki Yamada, Yoshifumi Matsumoto, Norio Okahara, Katsuhiko Mikoshiba

**Affiliations:** 1 Laboratory for Developmental Neurobiology, Brain Science Institute (BSI), RIKEN, Wako, Saitama, Japan; 2 Central Institute for Experimental Animals, Kawasaki, Kanagawa, Japan; 3 Japan Science and Technology Agency, International Cooperative Research Project and Solution-Oriented Research for Science and Technology, Calcium Oscillation Project, Wako, Saitama, Japan

## Abstract

We report a methodology to chronically record *in vivo* brain activity in the awake common marmoset. Over a month, stable imaging revealed macroscopic sensory maps in the somatosensory cortex and their underlying cellular activity with a high signal-to-noise ratio in the awake but not anesthetized state. This methodology is applicable to other brain regions, and will be useful for studying cortical activity and plasticity in marmosets during learning, development, and in neurological disorders.

The common marmoset (*Callithrix jacchus*) has emerged as an attractive non-human primate model for neuroscience research, due to (1) ease of handling with a small body size; (2) high reproductive rate and relatively short gestational period (~5 months); (3) accessibility of transgenic technology^1^. Based on these advantages, transgenic marmosets are being produced that carry genetic modifications implicated in neurological disorders (e.g. Parkinson’s disease and Alzheimer’s disease)^2^. In parallel, robust behavioral tasks have been developed to investigate sensory and cognitive functions of marmosets^3^,^4^,^5^,^6^,^7^. However, to gain cellular- and circuit- level insights into the plasticity of cortical networks underlying normal behaviors and disease states, an experimental technique to chronically track neuronal ensemble activity is required. Recently, *in vivo* Ca^2+^ imaging was applied to marmosets^8^, but the experiments were performed exclusively under anesthesia, which not only prevents the recording of behaviorally relevant neuronal activity, but also artificially modifies brain activity at the macroscopic^9^ and cellular levels^10^,^11^. Here we established, for the first time, multiscale macroscopic and cellular chronic imaging of brain activity in the awake marmoset, by combining a novel body fixation device, systematic acclimation training and subject screening based on their behavior.

## Results

**Figure 1:**
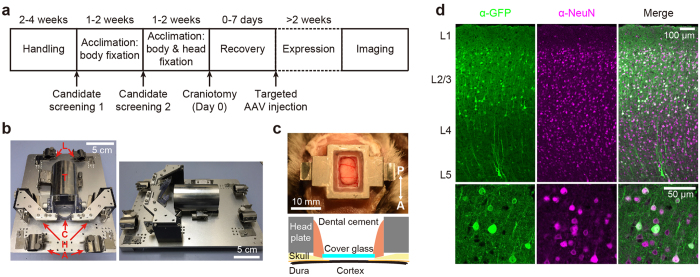
Experimental design for *in vivo* imaging with awake marmosets. (**a**) Experimental schedule. (**b**) Body fixation device for *in vivo* imaging (left: top view, right: side view). Parts for holding arms (‘A’), head (‘H’), chin (‘C’), trunk (‘T’), and legs (‘L’) are indicated. (**c**) An example image (top) and scheme (bottom) of cranial window. (**d**) Expression of GCaMP6s (‘α-GFP’), counter-staining of NeuN (‘α-NeuN’) and overlay of the two images (‘Merge’) with different magnifications (note the difference in scale bars).

In order to minimize motion artifacts during awake imaging, we developed a novel body fixation device, which holds the head, chin, arms, trunk, and legs of marmosets lying in a prone position (Fig. 1b and Supplementary Fig. 1; inspired by ref. 12). The space-saving configuration allows imaging under most commercially available microscopes. Marmosets were systematically subjected to handling and acclimation to the device for approximately a month, during which candidates were screened based on behavioral scores to assess their compatibility with the body fixation device (Online Methods, Supplementary Fig. 2, and Table 1). Subjects that experienced intensive acclimation training and showed good compatibility scores were implanted with a head post, and further acclimated to head and body fixation for an additional few weeks. A cranial window was then constructed based on stereotaxic coordinates^13^,^14^, and subregions responsive to sensory stimulation (identified with flavoprotein imaging, Supplementary Fig. 3) were targeted for virus injection to express a genetically encoded Ca^2+^ indicator, GCaMP6s^15^. Expression of GCaMP was specific to neurons (Fig. 1e; the percentage of NeuN+/GFP+ cells, 97.7 ± 0.6%, n = 5 sections from one animal), and was observed in more than one third of total neurons in a given infected area (Fig. 1e; the percentage of GFP+/NeuN+ cells, 36.5 ± 5.5%, n = 5 sections from one animal), which typically spanned over 500 μm per injection site.

**Figure 2:**
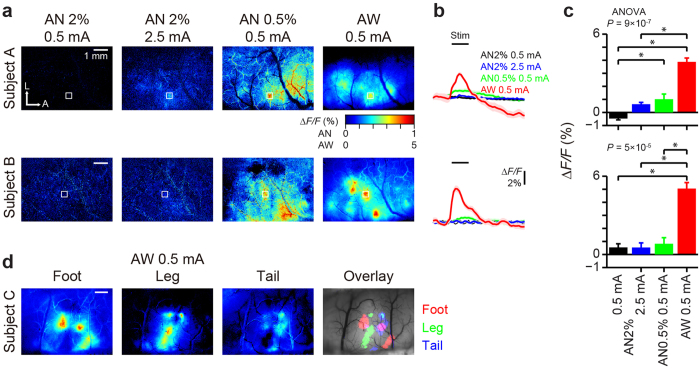
Macroscopic sensory responses in awake and anesthetized marmosets. (**a**) Macroscopic activity in the primary somatosensory cortex was recorded with epifluorescence microscopy under the anesthetized (AN) and awake (AW) states (subject A, day 17 and subject B, day 28). Responses to contralateral tactile stimulation were averaged across 10 trials, and the normalized fluorescent intensity changes (*ΔF/F*) of each pixel were averaged during stimulation and mapped with pseudo-color. (**b**) Traces of *ΔF/F* within ROIs (white boxes in **a**) upon stimulation (black bars; 1 s at 50 Hz) of the contralateral foot. Each trace is the mean across 10 trials, with shaded areas representing SEM. (**c**) Comparison of peak amplitude during stimulation derived from traces in **b**. Sensory responses were significantly stronger in the awake state than the anesthetized state (Kruskal-Wallis ANOVA; *P < 0.05 with Dunn’s post-hoc test). (**d**) Somatotopic representation of different body parts recorded in another subject (subject C, day 82). Pixels with *ΔF/F* values larger than 50% of the maximum in each condition were overlaid on the image of the cortex with different colors (‘Overlay’; responses to foot in red, leg in green and tail in blue).

Using well-trained marmosets expressing GCaMP, we performed macroscopic imaging of somatosensory cortex with epifluorescence microscopy, and investigated responses evoked by tactile stimulation (0.5 or 2.5 mA, 10 ms electrical pulses at 50 Hz for 1 s) to contralateral foot (Fig. 2a–c, subject A and B). Little or no activity pattern could be observed in the high anesthesia condition (2% isoflurane) even when higher stimulus intensity was tested (Fig. 2a–c), while sensory responses could be observed in the low anesthesia condition (0.5% isoflurane). In stark contrast, prominent responses were evoked by sensory stimulation in the awake condition (Fig. 2a–c). The difference of peak amplitudes between the brain states was substantial and statistically significant (Subject A: −0.5 ± 0.1%, 0.6 ± 0.1%, 1.0 ± 0.4%, 3.9 ± 0.3%; Subject B: 0.5 ± 0.3%, 0.5 ± 0.4%, 0.8 ± 0.5%, 5.1 ± 0.5% for AN2% 0.5 mA, AN2% 2.5 mA, AN0.5% 0.5 mA, and AW 0.5 mA, respectively). Comparable results were obtained with two other animals, with which only the high anesthesia condition was tested (Subject C and D, Supplementary Fig. 4). The spatial patterns of sensory responses in the low anesthesia condition were also somewhat different from those in the awake condition: activation in the posterior region, presumably corresponding to area 1, was more prominent in the awake condition than in the low anesthesia condition. These results are consistent with a previous report with intrinsic imaging in squirrel monkeys^16^ and fMRI in marmosets^9^. Stimulation of different body parts (foot, leg and tail) evoked responses in distinct subregions of the brain with partial overlap, which resulted in clear somatotopic maps (Fig. 2d and Supplementary Fig. 4).

**Figure 3:**
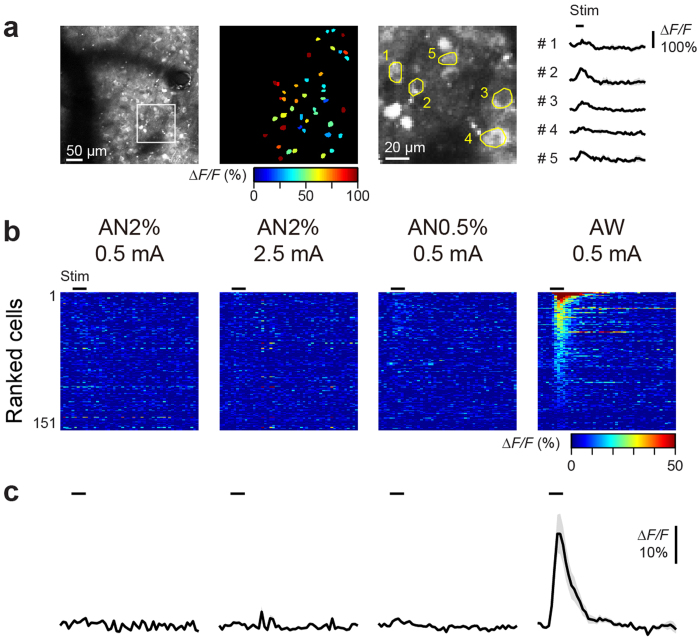
Cellular sensory responses in awake and anesthetized marmosets. (**a**) 2-photon imaging of GCaMP6s-expressing cells in the foot-responsive region identified by macroscopic imaging (subject C, day 101). Left image, an example field of view covering approximately 425 × 425 μm^2^. Middle image, stimulation-evoked activity maps of the field of view; each contour represents ROI overlaid on cell bodies, with pseudo-colored peak amplitude during stimulation. Right image, an expanded image of a white box in the left image with ROIs overlaid. The rightmost traces, *ΔF/F* within each ROI averaged across 4 trials, shown as mean ± SEM. Black bars represent stimulation period (1 s, 50 Hz). (**b**) Heat maps of activity from all cells (n = 151 cells from 2 animals; subject A on day 41 and B on day 32), ranked by the peak amplitude during stimulation period (AW foot 0.5 mA). (**c**) Traces of *ΔF/F* averaged across all the recorded cells, with shaded areas representing SEM.

We further examined the cellular activity of responsive somatosensory cortical subregions by 2-photon imaging (Fig. 3). Consistent with the macroscopic imaging, robust sensory responses were observed at cellular level in the awake condition (Fig. 3a,b), and there was a prominent difference between the awake condition and anesthesia condition (Fig. 3b,c): the percentage of responsive cells was much higher (AN2% 0.5 mA, 2.7 ± 1.8%; AN2% 2.5 mA, 1.6 ± 0.6%; AN0.5% 0.5 mA, 8.1 ± 5.3%; AW 0.5 mA, 64.1 ± 15.9%; n = 2 animals), and the peak amplitude was significantly larger in the awake condition (*ΔF/F*: AN2% 0.5 mA, 3.5 ± 0.7%; AN2% 2.5 mA, 1.7 ± 0.8%; AN0.5% 0.5 mA, 3.4 ± 0.7%; AW 0.5 mA, 27.7 ± 5.4%; Friedman’s ANOVA: *P* = 2 × 10^−27^, Dunn’s post-hoc test: *P* < 10^−4^ between AN vs AW; n = 151 cells from 2 animals). Comparable results were obtained with another animal, with which only the high anesthesia condition was tested (Supplementary Fig. 5d,e), both in terms of the percentage of responsive cells (AN2% 0.5 mA, 1.6%; AN2% 2.5 mA, 4.7%; AW 0.5 mA, 97.2%) and the peak amplitude (*ΔF/F*: AN2% 0.5 mA, 0.2 ± 0.5%; AN2% 2.5 mA, 5.0 ± 0.6%; AW 0.5 mA, 65.3 ± 2.1%). Taken together, these results reveal prominent artifacts of anesthesia on brain activity, and demonstrate the advantage of our awake recording methodology for the accurate investigation of sensory responses.

**Figure 4:**
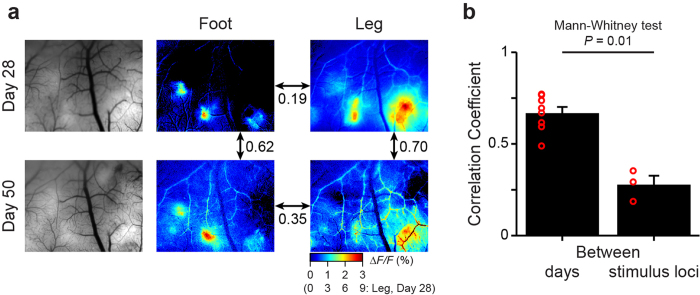
Longitudinal imaging of sensory maps in awake marmosets. (**a**) The cortical surface images with vasculature patterns (left) and pseudo-color response maps (middle: foot, right: leg) of subject D recorded on day 28 (top) and 50 (bottom). Correlation coefficients between the maps are shown with arrows. (**b**) Similarity of maps between days or stimulus locations. A correlation coefficient was calculated from a pair of maps acquired from the same stimulus location on different days (‘Between days’) or from the different stimulus locations on the same day (‘Between stimulus loci’). Data are represented as mean + SEM, and values from individual comparisons are shown with red circles.

Finally, we validated our system for chronic recording. The quality of the cranial window was regularly monitored and the brain surface was cleaned upon necessity to circumvent tissue regrowth (Methods). We imaged somatosensory responses in awake marmosets over weeks or months, and analyzed the stability of sensory representations (Fig. 4a for subject D and Supplementary Fig. 6a for subject C). We quantified the similarity between a pair of maps by calculating Pearson’s correlation coefficient between *ΔF/F* values of all pixels from each map (see Online Methods for detail). Despite variations in the peak amplitude across days, the similarity between a pair of maps representing the same body parts on different days was significantly higher than that between a pair of maps representing different body parts on the same days (Fig. 4b; 0.67 ± 0.03 for between days, 8 pairs from 2 animals; 0.28 ± 0.05 for between body parts, 3 pairs from 2 animals). This indicates that the somatotopic arrangement of sensory representations remained relatively stable over time.

We also performed chronic 2-photon imaging to analyze the stability of sensory responses at a cellular level (Supplementary Fig. 6b). Although there was a significant yet small difference in the peak amplitude between days (Day 32: 70.6 ± 42.0% vs Day 39: 66.0 ± 20.9%; Wilcoxon sign rank test: *P* = 0.03; n = 19 cells from 1 animal), the ensemble correlation (see Methods) between different days was close to that observed in macroscopic imaging (0.67 ± 0.13, mean ± SEM during stimulation period). Although the sample size was relatively limited due to technical difficulties (see Discussion), these data imply that the sensory responses may be also preserved at a cellular level.

## Discussion

Here we established a longitudinal imaging method with macroscopic and cellular resolution in the awake marmoset. The proof-of-principle experiments demonstrated a striking difference of neuronal Ca^2+^ signals depending on brain state, underscoring the importance of recording brain activity in awake animals, and successfully revealed the stability of sensory representations for over a month.

We believe that some of the excluded subjects might have been eligible for experiments, but we employed relatively stringent criteria for screening candidates given the high cost of animals, the demanding nature of the experiment that involves many surgical steps, and the high mechanical stability required for cellular imaging.

Using the conventional AAV system, a sufficient level of GCaMP expression for *in vivo* macroscopic and cellular imaging was achieved in our hands. For future experiments, it will be useful to enhance the expression level of GCaMP with the tetracycline-inducible AAV system that has been used with mice^17^,^18^ and was recently reported with marmosets^8^.

We noticed that the time course of sensory responses was qualitatively different between macroscopic imaging and cellular imaging, with the former occasionally reaching a peak within the stimulation period. Indeed, both time to peak and half decay were shorter in macroscopic imaging (0.33 ± 0.05 s and 0.46 ± 0.08 s, respectively; n = 8 regions from 4 animals) compared to 2-photon imaging (1.13 ± 0.04 s and 0.86 ± 0.02 s, respectively; n = 616 cells from 3 animals) (Wilcoxon rank sum test, *P* = 2 × 10^−6^ and 6 × 10^−3^). These results may be well explained by the notion that signals in macroscopic imaging are mostly originated from neuropils, where Ca^2+^ signals show faster dynamics than those of somata^19^,^20^,^21^.

Although we could chronically track some cells in the same field of view, the number of cells recorded was relatively limited in the current study. This is probably due to the cranial window reconstruction procedure, which likely changed the cellular geometry within the focal plane and/or the relative angle of the focal plane against the microscope objective. In the future, this might be resolved by the use of artificial dura^22^, the use of a stacked cover glass that fills the craniotomy more efficiently to prevent tissue regrowth^8^,^23^, or functional volume imaging that could record responses from cells that lie within multiple focal planes^24^,^25^.

Our experimental system can be readily applied to other cortical areas located on the surface of the brain, is compatible with behavioral tasks under head fixation^5^,^7^, and will be useful for studying sensorimotor plasticity in learning, development, and neurological disorders.

## Methods

### Subjects

All experimental procedures were performed in accordance with the regulations and guidelines of Central Institute for Experimental Animals (CIEA, Japan) and the Japan Neuroscience Society, and were approved by the Institutional Animal Care and Use Committee of CIEA (CIEA approval No. 14037). Adult common marmosets (*Callithrix jacchus*) obtained from Central Laboratory for Experimental Animals (CLEA Japan, Inc., Tokyo, Japan) were used in this study. Body weight of the animals was closely monitored throughout the experiment, and the experiment was either terminated or paused for intensive care until recovery when animals lost more than 10% of body weight or show signs of discomfort. The marmosets were individually housed in a temperature- and humidity-controlled animal facility (27 °C, 12-h light/dark cycles), and supplied with *ad libitum* water and balanced diet (CMS-1M; CLEA Japan. Inc.). All experiments were conducted during the daytime.

### Acclimation to body fixation and subject screening

We screened candidate subjects from adult marmosets (>2 years old) with standard body weight (300–400 g) (Table 1). They were first subjected to daily handling for two to four weeks, during which compatibility to the experiment was scored based on their behavior. We assessed (1) spontaneous aggressiveness, (2) aggressiveness during handling or fixation, (3) frequency of cries during handling or fixation, and (4) loudness of cries during handling or fixation with score ranging from 0 (none, weak or rare) to 2 (strong or frequent). The scores of the four items were summed, making the final score for each subject range between 0 (adaptive) to 8 (resistant). Based on the scores averaged across the last three days of handling (Table 1), several marmosets were next acclimated to the body fixation device for 9 days (Fig. 1a and Supplementary Fig. 2): marmosets were placed in a prone position to the custom-made body fixation device without head fixation for 5 min (day 1–3), 15 min (day 4–6) and 25 min (day 7–9) under room light. The behavior of the candidates was scored as above (Table 1), and four subjects (subject A–D) were selected for functional imaging and subjected to head plate implantation. One week after implantation, the marmosets were acclimated to head and body fixation for 15 min (day 1–3), 30 min (day 4–6) and 1 h (day 7–9) in the dark followed by cranial window surgery and targeted AAV injection.

### Cranial window surgery

Prior to surgery, marmosets were anesthetized with isoflurane (4% induction, 2% maintenance; Mylan Inc.). The level of anesthesia was routinely examined with withdrawal reflex to leg pinch, and the body temperature was maintained at ~37 °C using a heating blanket and a rectal thermal probe. Blood oxygen saturation and heart rate were monitored using pulse oximetry (SpO_2_). The hair on the scalp was trimmed, local anesthesia (2% xylocaine jelly; Astra Zeneca) was applied, and the skin was carefully removed after disinfection. A custom-made stainless steel head plate was glued to the skull using dental cement (Sun Medical) above an area covering the left somatosensory cortex according to the brain atlas^13^,^14^. Craniotomy (AP: −4 to +6 mm, ML: −1 to 6 mm from bregma) was performed with a dental drill, and the skull around the craniotomy was slightly thinned to snugly fit a cover glass onto the brain. The dura matter was carefully removed. A glass coverslip was placed on the brain tissue and glued to the skull with dental cement (Fig. 1b). Animals were allowed to recover for at least a week before targeted virus injection. Antibiotics were subcutaneously administered for three consecutive days after surgery (ampicillin sodium, 5 mg per animal, Meiji Seika Pharma CO., Ltd.). For chronic imaging, the cranial window was regularly checked and any tissue regrowth was carefully removed^23^.

### Targeted virus injection

The subregions in the somatosensory cortex responsive to tactile stimulation were functionally identified by flavoprotein imaging^26^ using an epifluorescence microscopy (THT Microscope, Brain Vision). The brain surface (8.6 × 6.6 mm) was illuminated with a blue LED (LEX2-B, Brain Vision; 425–475 nm), and fluorescence (500–550 nm) was recorded at 10 Hz by a cooled CCD camera (672 × 512 pixels after binning 2 × 2; ORCA-ER, Hamamatsu Photonics) with a 1 × objective lens (10450028, NA 0.44, Leica Microsystems). Electrical stimulation (50 Hz for 1s, 0.5–2.5 mA) was applied on the surface of the contralateral foot, leg or tail. The brain area identified to be responsive to foot stimulation by flavoprotein imaging under the awake state was targeted for virus injection; when no clear responses were observed, multiple locations devoid of large blood vessels were targeted (Supplementary Fig. 3). Marmosets were anesthetized with isoflurane and administered with mannitol (2 g/kg, I.P.) to prevent brain edema and to facilitate spread of virus solution^18^. The coverslip over the craniotomy was carefully removed, and AAV2/1-syn1-GCaMP6s-WPRE and AAV2/1-CB7-RFP-WPRE (Penn Vector Core) were co-injected at 30 nl/min up to 1 μl with a glass pipette (outer diameter ~30 μm; P-87, Sutter Instruments) connected to a pressure injector (IM 300 Microinjector, NARISHIGE) at 300 μm deep from the surface. The pipette was held in place for at least 5 min before removal, and a new coverslip was placed over the craniotomy. The marmosets were allowed to recover for at least 2 weeks before *in vivo* Ca^2+^ imaging. The timing of imaging is presented as the number of days from the cranial window surgery (day 0).

### *In vivo* Ca^2+^ imaging

Macroscopic imaging of GCaMP signal was performed with the same system used for flavoprotein imaging. Cellular imaging was performed with a two-photon laser-scanning microscope (LSM7 MP, ZEISS) equipped with a Ti:sapphire laser (Cameleon, COHERENT) and a water-immersion objective lens (20×, W Plan-Apochromat, NA 1.0, ZEISS). The laser wavelength was tuned to 1020 nm. Fields of view covering 425 × 425 μm at 100–300 μm from the surface were imaged at a spatial resolution of 256 × 256 pixels and at a frame rate of 6.7 Hz. Imaging under the anesthetized state (0.5 or 2% isoflurane) was performed after that under the awake state on the same days.

### Image analysis

Image analysis was performed with custom-written scripts in ImageJ and Matlab (MathWorks). Image stacks were corrected for in-focal (XY) plane brain motion using cross-correlation based on rigid body translation (StackReg plugin)^27^. The corrected stacks were manually inspected for out-of-focal (Z) plane brain motion, and stacks with motion artifacts were discarded from further analysis. Regions of interest (ROIs) were manually drawn over brain regions and cell bodies for macroscopic imaging and 2-photon imaging respectively, and average fluorescence intensity values within each ROI were extracted. We calculated *ΔF/F*, or the change in fluorescence relative to baseline (1 s prior to stimulation) divided by the mean of fluorescence during baseline. Responses during 2-photon imaging were considered significant when *ΔF/F* values during stimulation period exceeded twice the standard deviation of those during the baseline in trial-averaged traces.

Pseudo-color maps of somatosensory responses were created with the *ΔF/F* values averaged during the stimulation period and across trials. For quantifying the similarity of maps, Pearson’s correlation coefficient was calculated with *ΔF/F* values from all the pixels; only the data from foot and leg stimulation were used, since those from tail stimulation had low signal-to-noise ratio with a diffuse spatial distribution. Data were obtained from subject C on day 82 (foot and leg), 93 (foot), 102 (foot), and 136 (foot); subject D on day 28 (foot and leg) and 50 (foot and leg).

For quantifying the similarity of cellular responses, population vectors consisting of *ΔF/F* values from all the cells were constructed at each time point of imaging, and Pearson’s correlation coefficient was calculated between vectors derived from two different days.

### Immunohistochemistry

Marmosets were deeply anesthetized with ketamine hydrochloride (50 mg/kg, i.p.; Fujita Pharma), xylazine (4 mg/kg, i.p.; BAYER Yakuhin, Ltd) and isoflurane (2%). After transcardial perfusion of 4% paraformaldehyde, the somatosensory cortex was dissected out and postfixed overnight at 4 °C. Coronal sections (50 μm) were prepared with a vibratome. Slices were first rinsed with phosphate buffered saline (PBS) and nonspecific binding was blocked with 5% normal goat serum (Jackson ImmunoResearch) in PBS with 0.1% Triton X-100 for 1 h at room temperature before incubation with primary antibody (rat monoclonal anti-GFP, 1:500; Nacalai and mouse polyclonal anti-NeuN, 1:500; Clontech) in PBS with 0.01% Triton-X 100 overnight at 4 °C. The primary antibody was detected with an Alexa Fluor 488-conjugated goat anti-rat (1:1000; A-11006, Invitrogen) and Alexa Fluor 644-conjugated goat anti-mouse (1:1000; A-21052, Invitrogen) secondary antibody incubated for 1 h at room temperature. Sections were imaged with a confocal laser-scanning microscope (TCS SP5, Leica). The number of GFP-positive and NeuN-positive cells was semi-automatically counted (five sections from one animal).

## Additional Information

**How to cite this article**: Yamada, Y. *et al.* Chronic multiscale imaging of neuronal activity in the awake common marmoset. *Sci. Rep.* **6**, 35722; doi: 10.1038/srep35722 (2016).

## Supplementary Material

Supplementary Information

## Figures and Tables

**Table 1:** Information of subjects.

ID	Age (months)	Sex	Weight (g)	Behavioral score (0: adaptive; 8 resistant)	
				Handling	Acclimation: Body fix.
5328	17	♀	340	0.0	4.0
5387	22	♀	290	3.0	—
5825	25	♂	410	6.7	—
5329	25	♀	320	3.7	—
5370	27	♀	410	4.3	—
**5363 (D)**	27	♂	330	0.0	1.3
5362	27	♀	290	2.7	—
5342	28	♀	360	0.7	2.0
5097	36	♀	360	2.3	3.3
171	51	♂	310	4.3	—
351	53	♀	330	2.0	5.3
5038	54	♀	380	5.7	—
277	58	♀	370	5.7	—
4871	59	♀	380	2.7	4.3
4874	59	♀	370	2.7	5.3
245	60	♀	340	2.7	4.7
**4834 (B)**	60	♀	350	1.7	3.3
4844	60	♀	350	5.3	—
4850	60	♀	310	5.3	—
4809	61	♀	300	5.0	—
1012	62	♂	310	5.7	—
231	62	♂	310	5.0	—
5101	63	♂	310	4.3	—
5102	63	♂	320	5.0	—
4276	65	♀	390	6.7	—
4205	67	♀	380	3.7	—
4176	68	♀	390	4.0	—
**137 (A)**	69	♀	350	1.0	2.7
**3833 (C)**	85	♂	360	0.0	0.0
3826	89	♂	360	3.0	—
3525	90	♀	380	4.0	—
3512	90	♀	380	4.0	—
3062	110	♀	350	3.0	—

Institutional ID, age, sex, weight and behavioral score are shown. Data are sorted by the age of subjects at the beginning of screening. Behavioral scores were averaged during the last three days of each session, which could range from 0 (adaptive to handling/fixation) to 8 (resistant to handling/fixation). Four subjects used for functional imaging are highlighted in bold.
