# Supplementary material for: Chronic multiscale imaging of neuronal activity in the awake common marmoset
Source: Sci Rep. 2016 Oct 27;6:35722. doi: 10.1038/srep35722 (PMC5082371; doi:10.1038/srep35722)
Supplement: Supplementary Information [file srep35722-s1.doc]

**Supplementary Information for**

**Chronic multiscale imaging of neuronal activity in the awake common marmoset.**

Yoshiyuki Yamada^1,2,3,4^, Yoshifumi Matsumoto^1,2,4^, Norio Okahara^2^ and Katsuhiko Mikoshiba^1,2^

1. Laboratory for Developmental Neurobiology, Brain Science Institute (BSI), RIKEN, Wako, Saitama, Japan. 2. Central Institute for Experimental Animals, Kawasaki, Kanagawa, Japan. 3. Present address: Department of Basic Neurosciences, University of Geneva, Geneva, Switzerland. 4. These authors contributed equally to this work.

Correspondence should be addressed to Y.Y. ([yy@brain.riken.jp](mailto:yy@brain.riken.jp)) or K.M. (mikosiba@brain.riken.jp).


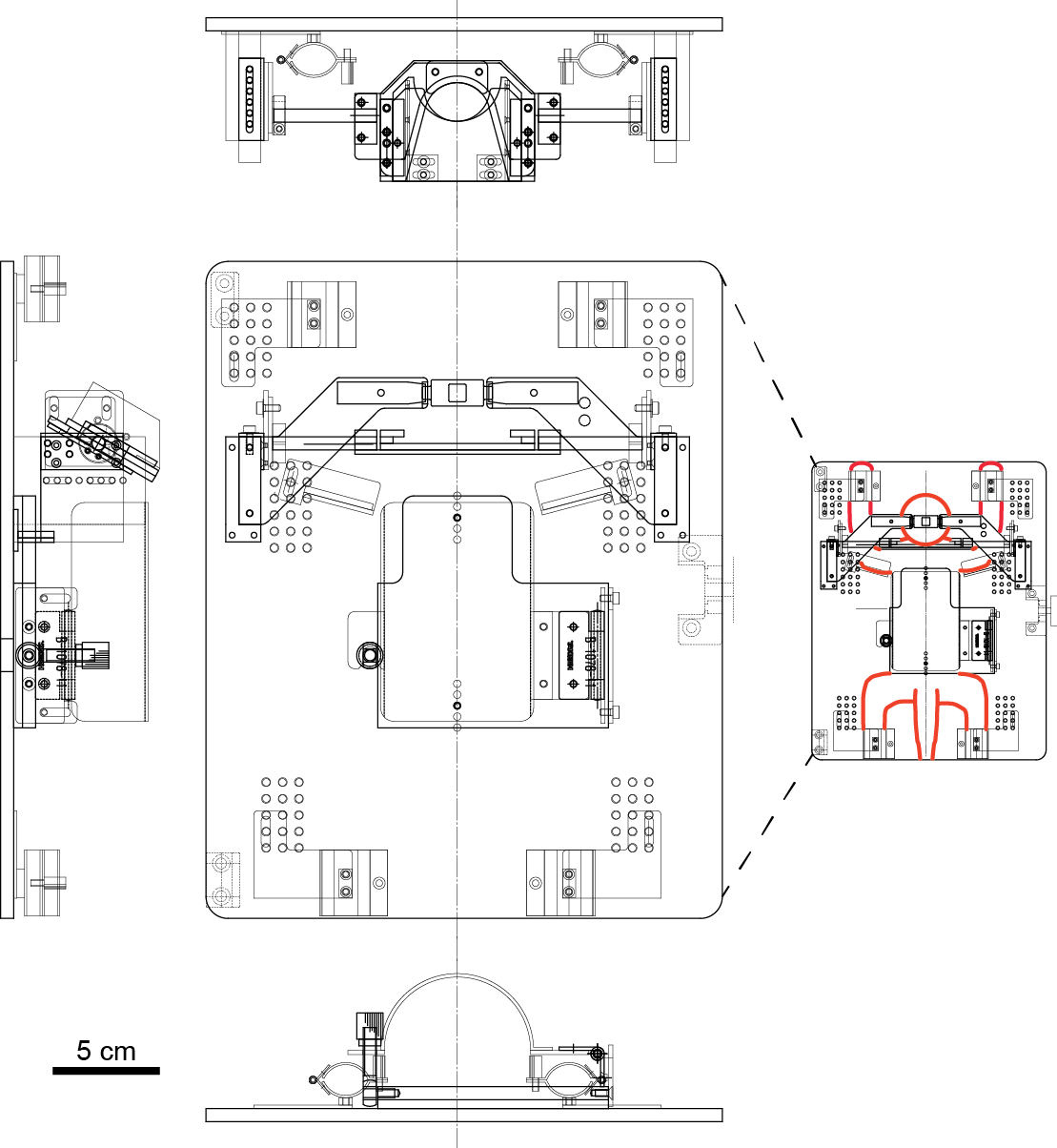


**Supplementary Figure 1** Detailed design of body fixation device.

**
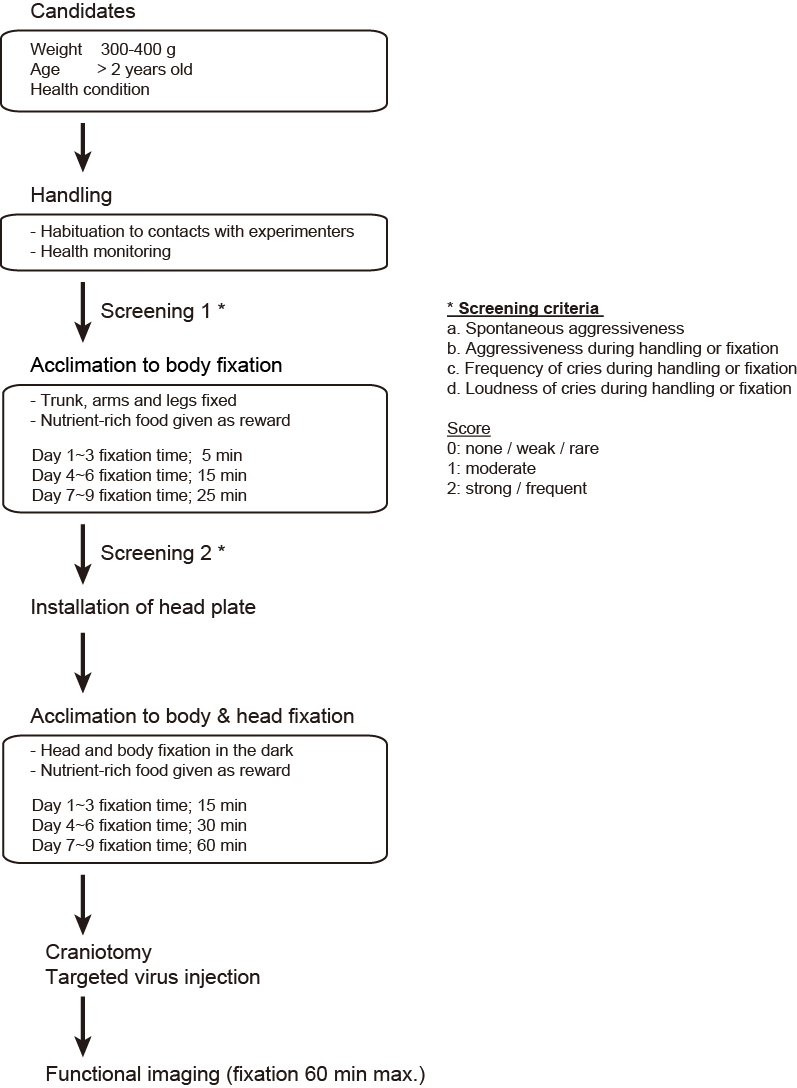
**

**Supplementary Figure 2** Flow chart of subject training and screening based on behavior scoring.

**
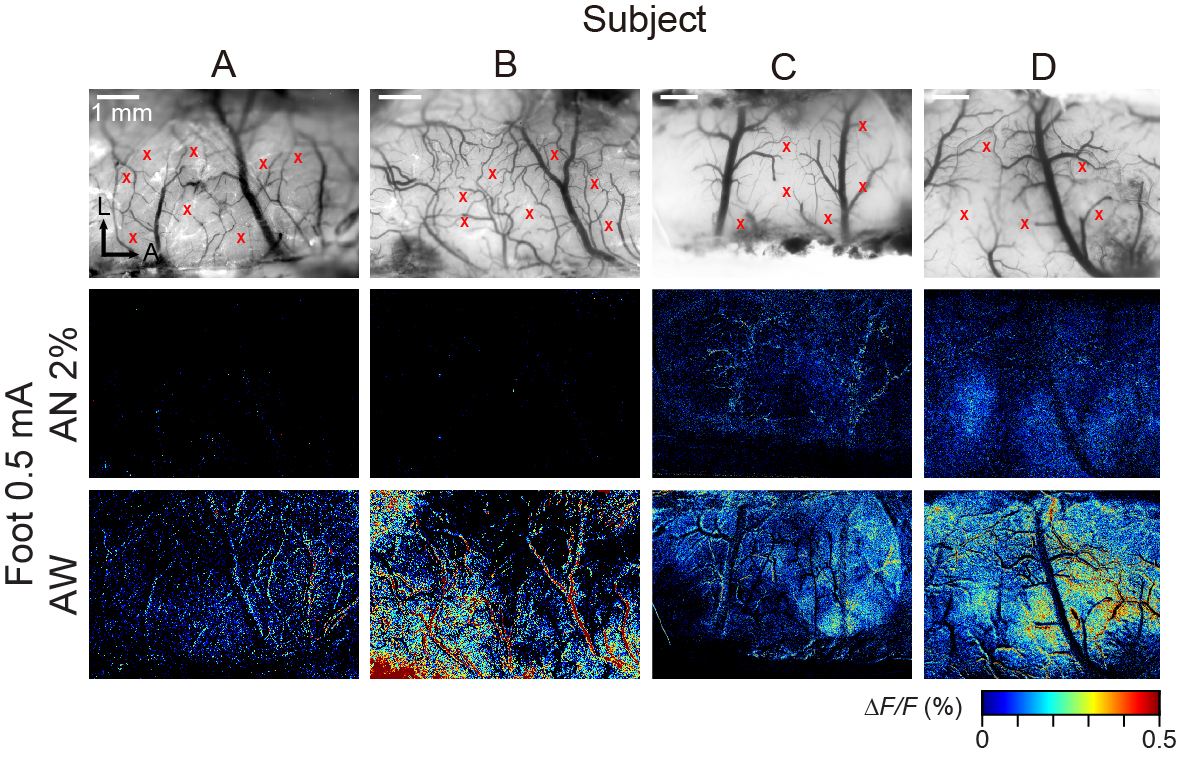
**

**Supplementary Figure 3** Identification of subregions responsive to sensory stimulation by flavoprotein imaging. Image stacks were averaged across 20 trials, and *ΔF/F* values of each pixel during stimulation period were averaged. The injection sites are indicated with red crosses.


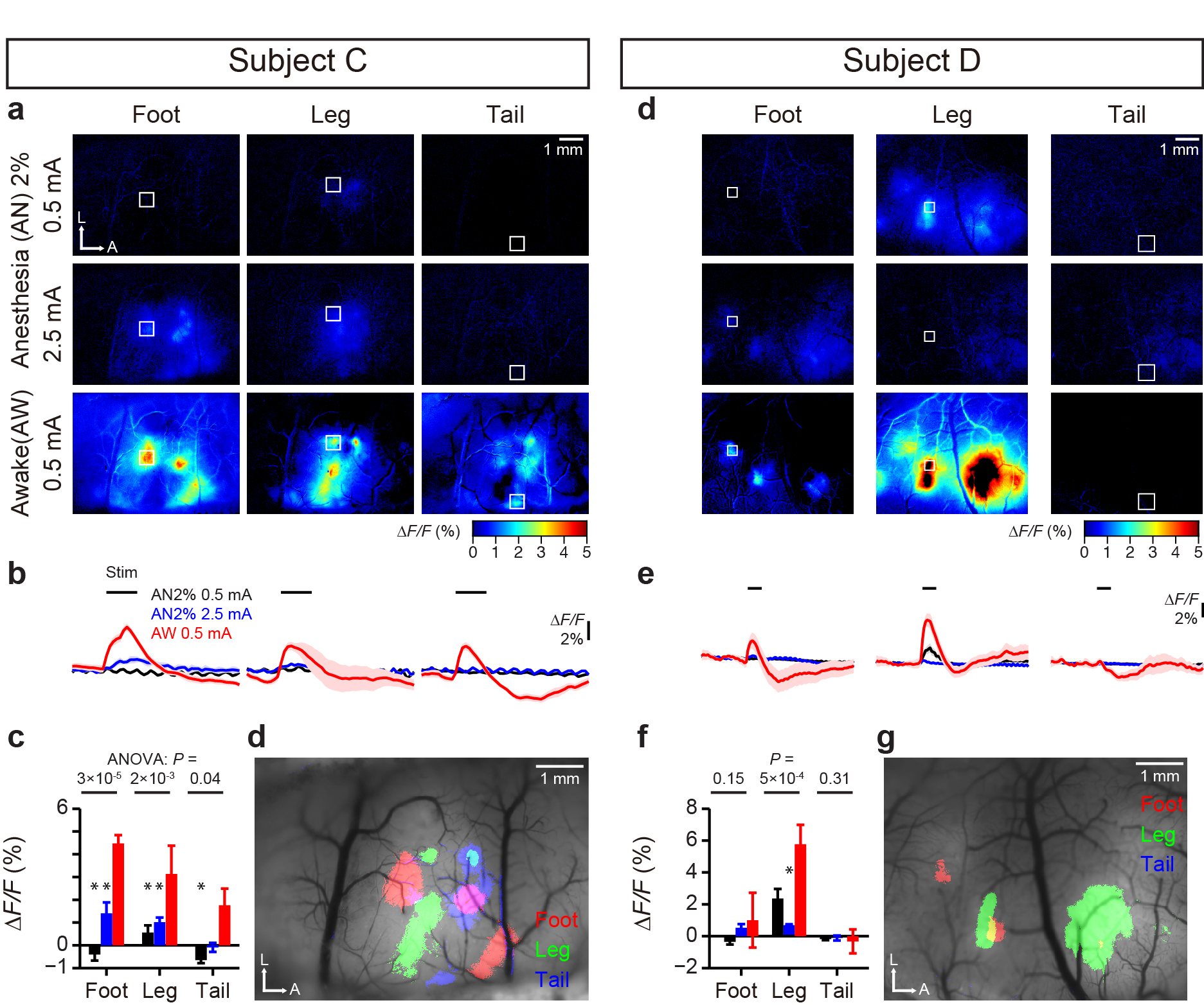


**Supplementary Figure 4** Sensory maps obtained from other subjects (subject C on day 82 and subject D on day 28). Data are shown as in **Fig. 2**. Some of the panels for subject C are the same as those presented in **Fig. 2d**.

**
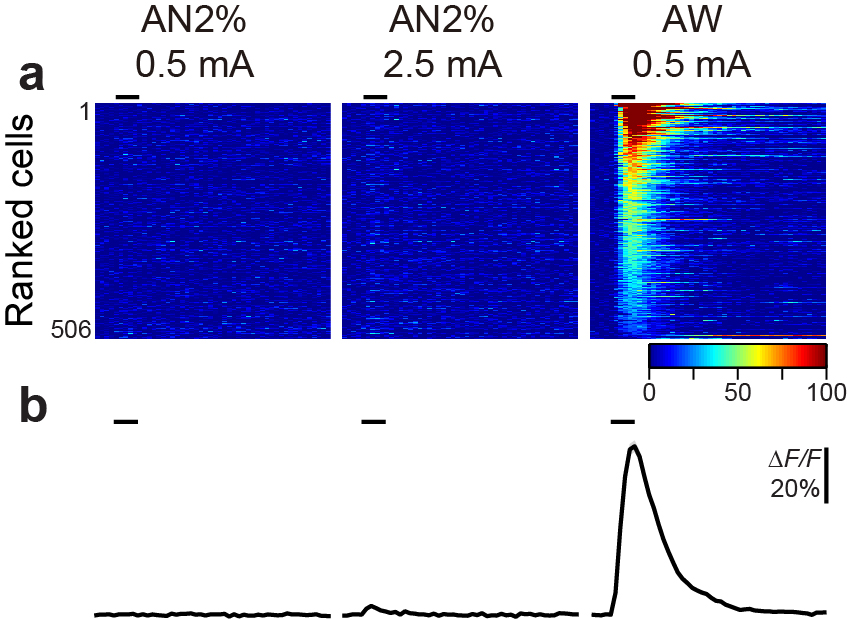
**

**Supplementary Figure 5** Cellular sensory responses in another subject (subject C, day 101) recorded in high anesthesia condition (2% isoflurane). (**a-b**) 2-photon imaging data acquired with foot stimulation (n = 506 cells). Data are presented as in **Fig. 3b-c**.


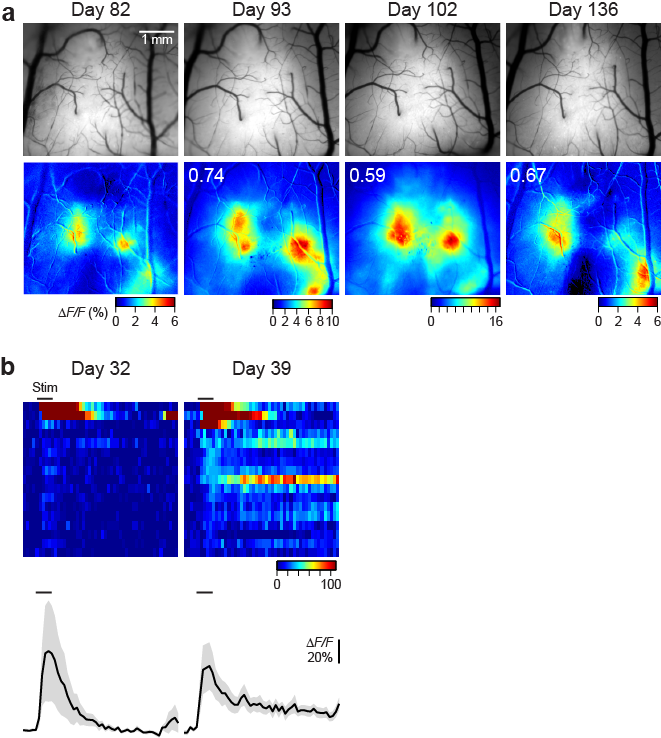


**Supplementary Figure 6** Stability of sensory responses. (**a**) The cortical surface images with vasculature patterns (top) and pseudo-color maps (bottom, 0.5 mA foot stimulation under awake state) recorded from subject C on day 82, 93, 102 and 136. The correlation coefficient between the map on day 82 and that on another day is indicated on the upper left corner in white. (**b**) The chronic 2-photon imaging data from subject B. Top: Heat maps of activity from all cells (n = 19 cells) ranked by the peak amplitude during stimulation period (black bars; 1 s at 50Hz, AW foot 0.5 mA). Bottom: responses averaged across all the recorded cells. Shaded areas represent SEM.
